# Opportunistic infections in immunosuppressed patients with juvenile idiopathic arthritis: analysis by the Pharmachild Safety Adjudication Committee

**DOI:** 10.1186/s13075-020-02167-2

**Published:** 2020-04-07

**Authors:** Gabriella Giancane, Joost F. Swart, Elio Castagnola, Andreas H. Groll, Gerd Horneff, Hans-Iko Huppertz, Daniel J. Lovell, Tom Wolfs, Troels Herlin, Pavla Dolezalova, Helga Sanner, Gordana Susic, Flavio Sztajnbok, Despoina Maritsi, Tamas Constantin, Veronika Vargova, Sujata Sawhney, Marite Rygg, Sheila K. Oliveira, Marco Cattalini, Francesca Bovis, Francesca Bagnasco, Angela Pistorio, Alberto Martini, Nico Wulffraat, Nicolino Ruperto, for the Paediatric Rheumatology International Trials Organisation (PRINTO), Ruben Cuttica, Stella Maris Garay, Jurgen Brunner, Wolfgang Emminger, Simone Appenzeller, Claudio Len, Claudia Saad Magalhaes, Albena Telcharova-Mihaylovska, Miroslav Harjacek, Marija Jelusic, Anne Estmann, Susan Nielsen, Cristina Herrera Mora, Elisabeth Gervais, Isabelle Koné-Paut, Pierre Quartier, Ivan Foeldvari, Gerd Horneff, Thomas Lutz, Kirsten Minden, Nikolay Tzaribachev, Maria Trachana, Elena Tsitsami, Olga Vougiouka, Ilonka Orban, Liora Harel, Philip Hashkes, Yosef Uziel, Rolando Cimaz, Adele Civino, Rita Consolini, Gianfranco D’Angelo, Fabrizio De Benedetti, Giovanni Filocamo, Elena Fueri, Romina Gallizzi, Maria Cristina Maggio, Maria Greca Magnolia, Angela Miniaci, Davide Montin, Alma Nunzia Olivieri, Serena Pastore, Donato Rigante, Francesco Zulian, Ingrida Rumba-Rozenfelde, Valda Stanevicha, Violeta Panaviene, Ana Luisa Rodriguez Lozano, Nadina Rubio-Perez, Gabriel Vega Cornejo, Esther Hoppenreijs, Sylvia Kamphuis, Berit Flato, Ellen Berit Nordal, Reem Abdwani, Tatiana Miraval, Maria Eliana Paz Gastanaga, Elzbieta Smolewska, Constantin Ailioaie, Alexis-Virgil Cochino, Matilda Laday, Calin Lazar, Ekaterina Alexeeva, Vyacheslav Chasnyk, Vladimir Keltsev, Wafaa Mohammed Saad Suwairi, Gordana Vijatov-Djuric, Jelena Vojinovic, Thaschawee Arkachaisri, Elena Koskova, Tadej Avcin, Mahmood Ally, Christa Janse Van Rensburg, Ingrid Louw, Jordi Anton Lopez, Alina Lucica Boteanu, Inmaculada Calvo Penades, Jaime De Inocencio, Pablo Mesa-del-Castillo, Estefania Moreno, Agustin Remesal, Michael Hofer, Faysal Gok, Seza Ozen, Athimalaipet Ramanan, Chiara Pallotti, Luca Villa

**Affiliations:** 1 IRCCS Istituto Giannina Gaslini, Clinica Pediatrica e Reumatologia, PRINTO, Genoa, Italy; 2 Department of Pediatric Immunology and Rheumatology, Wilhelmina Children’s Hospital, University Medical Center Utrecht, University Utrecht, European Reference Network-RITA, Utrecht, The Netherlands; 3 Department of Infectious Diseases, IRCCS Istituto Giannina Gaslini, Genoa, Italy; 4 Infectious Disease Research Program, Department of Pediatric Hematology and Oncology, University Children’s Hospital, Münster, Germany; 5 Asklepios Clinic Sankt Augustin, Department of General Paediatrics, Sankt Augustin, Germany; 6 Medical Faculty, Department of Paediatric and Adolescents Medicine, University Hospital of Cologne, Cologne, Germany; 7 Clinic Bremen-Mitte, Prof.-Hesse Children’s Hospital and Pediatric Intensive Care Medicine, Bremen, Germany; 8 Division of Rheumatology, Cincinnati Children’s Hospital Medical Center, Cincinnati, OH USA; 9 Pediatric Rheumatology Unit, Aarhus University Hospital, Aarhus, Denmark; 10 1st Faculty of Medicine, Department of Pediatrics and Adolescent Medicine, Charles University in Prague and General University Hospital, Praha, Czech Republic; 11 Department of Rheumatology, Oslo University Hospital, Oslo, Norway; 12 Norwegian National Advisory Unit on Rheumatic Diseases in Children and Adolescents, Oslo, Norway; 13 Institute of Rheumatology of Belgrade, Division of Pediatric Rheumatology, Belgrade, Serbia; 14 Hospital Universitario Pedro Ernesto, Nucleo de Estudos da Saúde do Adolescente, Universidade do Estado do Rio de Janeiro, Rio de Janeiro, Brazil; 15 2nd Department of Pediatrics Athens Medical School, National and Kapodistrian University of Athens (NKUA), Athens, Greece; 16 Unit of Pediatric Rheumatology-Immunology, Second Department of Pediatrics, Semmelweis University, Budapest, Hungary; 17 Faculty of Medicine, Department of Paediatrics and Adolescent Medicine, Pavol Jozef Šafárik University in Košice, Kosice, Slovakia; 18 Sir Ganga Ram Hospital Marg, Centre for Child Health, Sir Ganga Ram Hospital, New Delhi, India; 19 Department of Clinical and Molecular Medicine, Faculty of Medicine and Health Sciences, NTNU - Norwegian University of Science and Technology, Trondheim, Norway; 20 Department of Pediatrics, St. Olavs University Hospital of Trondheim, Trondheim, Norway; 21 Instituto de Puericultura e Pediatria Martagao Gesteira (IPPMG), Universidade Federal do Rio de Janeiro, Rio de Janeiro, Brazil; 22 Clinica Pediatrica dell’Università di Brescia, Spedali Civili, Unità di Immunologia e Reumatologia Pediatrica, Brescia, Italy; 23 IRCCS Istituto Giannina Gaslini, Servizio di Epidemiologia e Biostatistica, Genoa, Italy; 24 Dipartimento di Neuroscienze, Riabilitazione, Oftalmologia, Genetica e Scienze Materno-Infantili (DiNOGMI), Università degli Studi di Genova, Genoa, Italy

**Keywords:** Infections, Opportunistic, Juvenile idiopathic arthritis, Immunosuppressive therapy, Biologics

## Abstract

**Background:**

To derive a list of opportunistic infections (OI) through the analysis of the juvenile idiopathic arthritis (JIA) patients in the Pharmachild registry by an independent Safety Adjudication Committee (SAC).

**Methods:**

The SAC (3 pediatric rheumatologists and 2 pediatric infectious disease specialists) elaborated and approved by consensus a provisional list of OI for use in JIA. Through a 5 step-procedure, all the severe and serious infections, classified as per MedDRA dictionary and retrieved in the Pharmachild registry, were evaluated by the SAC by answering six questions and adjudicated with the agreement of 3/5 specialists. A final evidence-based list of OI resulted by matching the adjudicated infections with the provisional list of OI.

**Results:**

A total of 772 infectious events in 572 eligible patients, of which 335 serious/severe/very severe non-OI and 437 OI (any intensity/severity), according to the provisional list, were retrieved. Six hundred eighty-two of 772 (88.3%) were adjudicated as infections, of them 603/682 (88.4%) as common and 119/682 (17.4%) as OI by the SAC. Matching these 119 opportunistic events with the provisional list, 106 were confirmed by the SAC as OI, and among them infections by herpes viruses were the most frequent (68%), followed by tuberculosis (27.4%). The remaining events were divided in the groups of non-OI and possible/patient and/or pathogen-related OI.

**Conclusions:**

We found a significant number of OI in JIA patients on immunosuppressive therapy. The proposed list of OI, created by consensus and validated in the Pharmachild cohort, could facilitate comparison among future pharmacovigilance studies.

**Trial registration:**

Clinicaltrials.gov NCT 01399281; ENCePP seal: awarded on 25 November 2011.

## Background

With the advent of biologic disease-modifying anti-rheumatic drugs (DMARDs), in a chronic condition like juvenile idiopathic arthritis (JIA), regulatory authorities such as the Food and Drug Administration (FDA) and the European Medicines Agency (EMA) have demanded from pharmaceutical companies and clinical researchers to evaluate the long-term safety of drugs used in children enrolled in phase II–III clinical trials [1–16]. Due to the limited number of patients enrolled in these trials [17], clinical researchers have devoted their work to the implementation of national and international registries [18–28] or to the analysis of insurance claim data [29–31].

During their development, all children experience a natural rate of infections compared to adults. Treatments in JIA with synthetic and biologic DMARDs are expected to increase the frequency of common infections and the risk of serious and opportunistic infections (OI) [23, 30–34], including especially tuberculosis in some geographic areas [35–37]. In order to tackle the long-term safety and efficacy evaluations, the Paediatric Rheumatology INternational Trials Organization (PRINTO) started in 2011 the “Pharmacovigilance in Juvenile Idiopathic Arthritis patients” (Pharmachild), an observational international registry supported by a European Union grant [38, 39].

Recent literature seems to confirm the high incidence of infections among JIA patients treated with immunosuppressants [21], but conclusive data are not available, yet. In particular, little evidence exists about the role of JIA or its immunosuppressive therapy in acquiring OI.

Several studies in the literature have the objective to define and classify OI, for example in HIV or in cancer patients [40–43]. In rheumatology, Winthrop and colleagues [32] were the first to convene a consensus meeting in 2015 to review the published literature on OI in patients with immune-mediated diseases treated with biologic DMARDs, in order to provide consensus recommendations for their evaluation in the context of clinical trials and observational studies.

Primary objectives of the present study were to derive a consensus-based list of opportunistic pathogens for use in children with JIA and confirm its role in identifying OI through the evaluation of the infectious events reported in Pharmachild registry by an independent Safety Adjudication Committee (SAC).

## Methods

### Pharmachild

The Pharmachild registry (project number 260353) involves 86 participating PRINTO (www.printo.it) centers in 32 countries [38] and the Paediatric Rheumatology European Society (PRES at www.pres.eu), with the aim to (1) monitor children with JIA for disease activity and comorbidity; (2) compare the long-term incidence rates of moderate, severe, and very severe adverse events (AE) and serious AE (SAE); and (3) assess the long-term efficacy of biologic and synthetic DMARDs in JIA. The Pharmachild registry contains both a retrospective and a prospective cohort. In brief, the retrospective cohort includes data from patients under treatment or previously treated with DMARDs obtained by one-time clinical chart review for safety events and complete drug exposure since disease onset to last available follow-up; the prospective cohort includes all cases newly treated with DMARDs since enrollment in the registry and cases still under treatment with any drug. In case of repeated events (e.g., infection with multiple reporting in the registry for the follow-up evaluation), only the initial event was considered. Full details of the registry methodology are available elsewhere [39].

### Study design

The study was divided into 5 main steps (additional figure 1).

#### Step 1: Provisional listing of opportunistic pathogens/infection presentations

The study Steering Committee (SC) included two PhD medical doctors (GG and JS), two certified Medical Dictionary for Regulatory Activities (MedDRA) coders (CP, LV), 3 biostatisticians (AP, FB, FB), and two Senior researchers (NW, NR).

The SAC was organized as an independent group of 5 physicians: 2 pediatric infectious disease specialists (EC and AG) and 3 pediatric rheumatologists (GH, HIH, DL), who have experience and expertise in the diagnosis and treatment of children with infectious or rheumatic diseases.

The SC starting point was the prior work by Winthrop et al. [32], an international consensus committee (infectious disease, public health, and pulmonary physicians and rheumatologists) that recommended a list of definite and probable OI after systematic review of literature on immune-mediated disorders (including JIA), and a consensus process. This list was discussed, modified, and approved by consensus by the SAC, through three subsequent Delphi web rounds, with the final result of a list of opportunistic pathogens/presentations for use in immunosuppressed children with JIA. In the first round, SAC members worked independently from each other, while during the second round, they could also revise their responses based on the review of comments from the other members. Final consensus was reached through a dedicated teleconference (moderator NR).

The SC then integrated the review of the literature with more recent evidence on OI in JIA [31, 44, 45] and prepared a provisional list of OI pathogens, then matched them with the MedDRA Preferred Terms (PT) in order to retrieve correctly the cases from the Pharmachild database. This provisional list was not shared with the SAC members as it was used only for data retrieval (see next step).

#### Step 2: Retrieval of infections in Pharmachild

For the Pharmachild study, the treating physicians reported online in the registry database all AEs from the disease onset to the last available follow-up visit. All terms contained in the MedDRA System Organ Class (SOC) “Infections and infestations” were considered in Pharmachild as Events of Special Interest (ESI) and classified in two different ESI sub-groups, named “tuberculosis” and “targeted infections (Epstein-Barr virus, cytomegalovirus, papilloma virus, herpes zoster primary and reactivation, and opportunistic infections).”

According to the Pharmachild protocol, all events (AEs and ESIs) of at least moderate intensity and all SAEs were collected. AEs and ESIs were coded initially by the treating physicians during data entry using the MedDRA dictionary, then recoded, if needed, by PRINTO-certified MedDRA coders and revised by the PRINTO medical monitor (JS), based on the most current version of MedDRA.

**Fig. 1:**
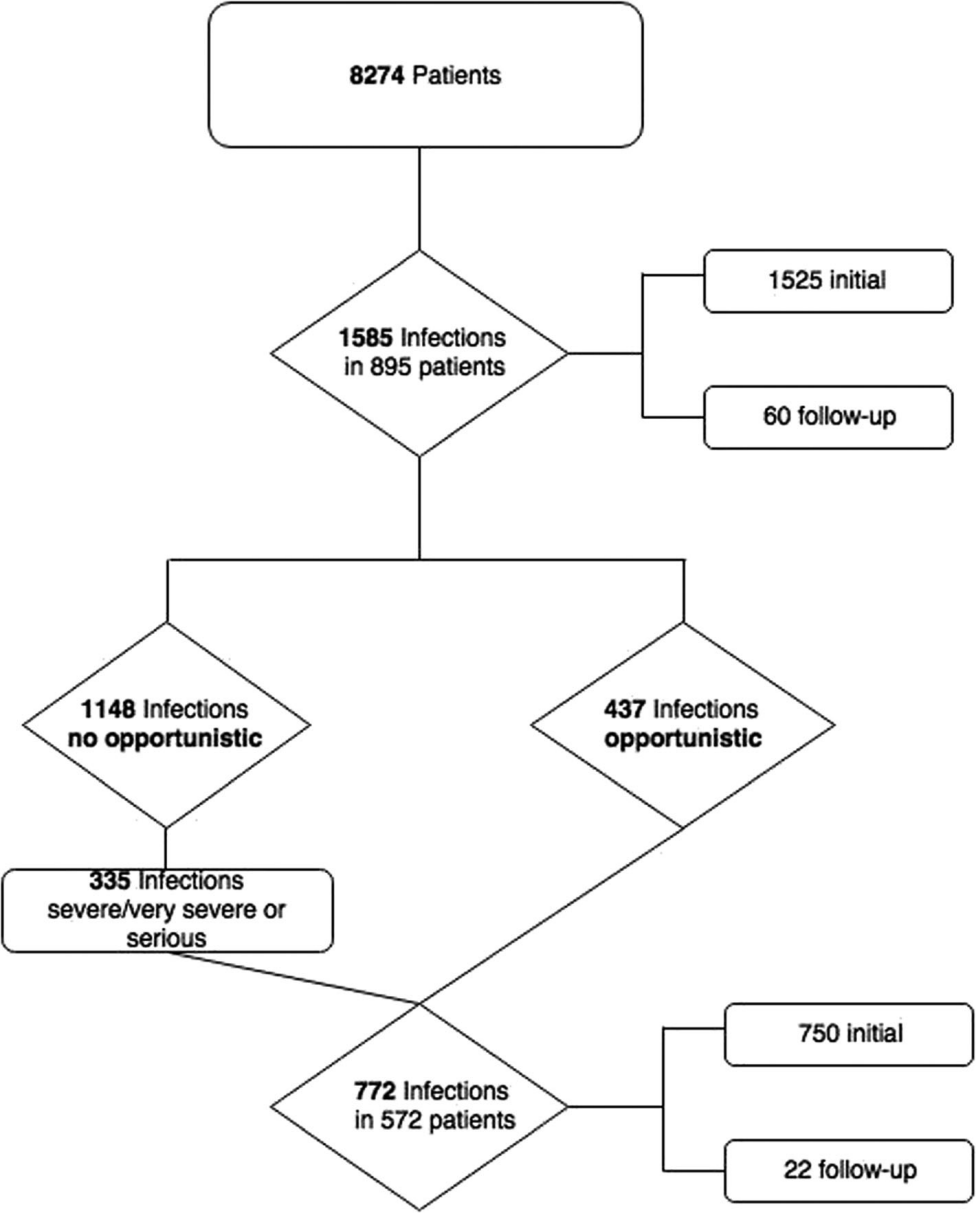
Flowchart of the Pharmachild population with infectious events

All infectious events (both initial and follow-up) in the MedDRA system organ class (SOC) (additional figure 2) “Infection and infestations” in Pharmachild as of January 2017 were retrieved (Fig. 1).

#### Step 3: Adjudication of infections by the SAC

A standard operating procedure (SOP) described the work to be done by the SAC. In brief, the SAC adjudication process included all the opportunistic events in the provisional list of OI derived by step 1 (any grade of severity) plus the non-OI infections of at least severe intensity and all serious infections from both retrospective and prospective charts.

The list of the events to be adjudicated by the SAC was provided in a dedicated external area of the PRINTO/Pharmachild website, with access through secure personal username and password.

The SAC members who reviewed all eligible cases (presented in numerical order by patient’s code) did not participate in the data collection in Pharmachild.

The complete patients’ data were available for the SAC members: (1) demographic characteristics of the patient (with personal data encrypted), (2) ILAR category of JIA, (3) laboratory and clinical information, (4) complete drug therapy with whole drug exposure for synthetic and biologic DMARDs since disease onset to the last available observation, (5) concurrent medications at the time of the infectious event, and (6) full AE report plus ESI-specific form for infections. In addition, JIA disease activity and a damage measure were available for prospective visits. The SAC members had the possibility to access the complete clinical information in a read-only mode, with no possibility to modify the original data. A numeric code, without any patient or center identifier and no a priori categorization of AE as OI, was provided to decrease potential bias during the adjudication exercise.

The SAC mandate was to evaluate each infectious case, based on the whole patient’s history available in Pharmachild, by answering 5 questions: (1) Based on the information provided, do you confirm that this patient had an infection?; (2) Is this infection common?; (3) Is this an opportunistic infection?; (4) Was the treatment appropriate for the infection?; (5) Could the event be possibly related to any of the drug(s) taken at the time of the event? The study SC was available to provide any additional information related to the event and required by the SAC at any time.

The consensus among the SAC members was defined as an agreement of at least 3 out of 5 (60%) members, on the first 3 out of 5 adjudication questions (“Is this an infection?,” “Is it common?,” “Is it opportunistic?”). Initially, the SAC members worked independently from each other, while in the next phase, for all cases without consensus, each member could access the evaluations of the other SAC members.

#### Step 4: Analysis of the Pharmachild registry

Step 4 was designed to evaluate, in an evidence-based fashion, the frequency of those events in the Pharmachild registry classified as infections by consensus among the SAC and to assign a final MedDRA code (High Level Term (HLT)/PT) to the event. In case of discrepancies in the categorization, after PRINTO and medical monitor (JS) check, a third independent examiner (GG) re-evaluated the individual case and assigned the final MedDRA code (HLT/PT).

#### Step 5: Final evidence-based listing of opportunistic pathogens/infection presentations

In this step, all the infectious events adjudicated by the majority of the SAC in Pharmachild were matched by MedDRA PT term with the provisional list of OI (see step 1) and divided in three groups: “confirmed OI,” if there was full agreement between the SAC and the provisional list of OI; “confirmed non-OI,” for the events adjudicated as non-OI by the SAC and missing in the provisional list; “possible/patient and/or pathogen-related OI,” for the remaining events in Pharmachild that could be possibly considered opportunistic depending on the physician’s evaluation of the patient history and by the detection of the specific pathogen causing the disease.

#### Statistical analysis

Descriptive statistics were reported in terms of absolute frequencies and percentages for qualitative data. Quantitative data were described in terms of median values and inter-quartile range (IQR) values due to their non-normal (Gaussian) distribution.

## Results

### Step 1: Provisional list of opportunistic pathogens/presentations

After the three web Delphi rounds, the probable and definite definitions of OI were agreed with one major change by 5/5 (100%) of the SAC [32]. In particular, the definition of definite OI was confirmed, while for probable infections, it was integrated with the following: “In case of the unusually severe course of infection due to a common pathogen with usually mild disease the pathogen might tentatively be considered opportunistic in a patient with impaired immune function.” Two definite categories of pathogens/presentations were modified by the SAC, while twelve were added in the provisional list of probable OI from the literature and matched with the HLT/PT MedDRA dictionary; none of the infections already included in the list by Winthrop et al. [33] was removed.

Table 1 shows the provisional list of pathogens/presentations, with the corresponding HLT terms according to MedDRA dictionary.

**Table 1:** Provisional list of pathogens/presentations and MedDRA HLT term approved by consensus by the SAC

Opportunistic infections definitions/pathogens	MedDRA HLT
**Definition of definite opportunistic infection in children with JIA**	
1. Generally does not occur in the absence of immunosuppression and whose presence suggests a severe alteration in host immunity **OR**	
2. Can occur in patients without recognized forms of immunosuppression, but whose presence indicates a potential or likely alteration in host immunity	
**List of definite pathogens and/or presentations of specific pathogens**	
Aspergillosis (invasive disease only)	Aspergillus infections
Bartonellosis (disseminated disease only)	Bartonella infections
BK virus disease including PVAN	BK virus infection
Blastomycosis	Blastomyces infections
Candidiasis (invasive disease or pharyngeal)	Candida infections
Coccidioidomycosis	Coccidioides infections/Paracoccidioides infections
Cryptococcosis	Cryptococcal infections
**Cytomegalovirus disease with onset at age > 1 month: pneumonia (CMV in BAL), colitis, CNS disease (CMV in CSF), liver (biopsy), retina (confirmed by ophthalmologist), nephritis, myocarditis, pancreatitis**	Cytomegaloviral infections
HBV reactivation	Hepatitis viral infections
Herpes simplex (invasive disease only)	Herpes viral infections
Herpes zoster (any form)	Herpes viral infections
Histoplasmosis	Histoplasma infections
Legionellosis	Legionella infections
*Listeria monocytogenes* (invasive disease only)	Listeria infections
Nocardiosis	Nocardia infections
Non-tuberculous mycobacterium disease	Atypical mycobacterial infections
Other invasive fungi: Mucormycosis (zygomycosis) (*Rhizopus*, *Mucor* and *Lichtheimia*), *Scedosporium* /*Pseudallescheria boydii*, *Fusarium*	Fungal infections NEC
*Pneumocystis jirovecii*	Pneumocystis infections
Post-transplant lymphoproliferative disorder (EBV)	Epstein-Barr viral infections
Progressive multifocal leucoencephalopathy	Polyomavirus infections
Salmonellosis (invasive disease only)	Salmonella infections
Strongyloides (hyperinfection syndrome and disseminated forms only)	Nematode infections
**Toxoplasmosis of central nervous system, onset at age ≥ 1 month; Disseminated toxoplasmosis, visceral toxoplasmosis**	Toxoplasma infections
Tuberculosis	Tuberculous infections
**Definition of probable opportunistic infection**	
Published data is currently lacking, but expert opinion believes that risk is likely elevated in the setting of DMARD therapy. In case of the unusually severe course of infection due to a common pathogen with usually mild disease the pathogen might tentatively be considered opportunistic in a patient with impaired immune function. Below there is a non-exhaustive list of possible pathogens	
**List of probable pathogens and/or presentations of specific pathogens**	
Campylobacteriosis (invasive disease only)	Campylobacter infections
*Cryptosporidium* species (chronic disease only)	Cryptosporidia infections
**Enterovirus chronic encephalitis**	Enteroviral infections NEC
**Giardia, Isospora: chronic (> 1 month) diarrhea**	Giardia infections/Isospora infections
HCV progression	Hepatitis viral infections
**Human Herpes Virus (HHV6–7): pneumonia, encephalitis**	Herpes viral infections
**Human Herpes Virus (HHV8): kaposi sarcoma**	Herpes viral infections
**Human metapneumovirus (hMPV): pneumonia, ARDS**	Viral infections NEC
**Human Papilloma Virus (HPV): extensive warts**	Papilloma viral infections
**Human respiratory syncytial virus (RSV): pneumonia with onset > 6 months of age**	Respiratory syncytial viral infections
Legionellosis	Legionella infections
Leishmaniasis (Visceral only)	Leishmania infections
Microsporidiosis	Protozoal infections NEC
**Molluscum contagiosum: chronic, disseminated**	Molluscum contagiosum
Paracoccidioides infections	Paracoccidioides infections
**Parvovirus B19: pure red cell aplasia**	Parvoviral infections
*Penicillium marneffei*	Fungal infections NEC
**Rota-Arena-Norovirus: chronic (> 1 month) diarrhea**	Rotaviral infections/Arenaviral infections/Caliciviral infections
Shigellosis (invasive disease only)	Shigella infections
*Sporothrix schenckii*	Sporothrix infections
Trypanosoma cruzi infection (Chagas’ disease) (disseminated disease only)	Trypanosomal infections
**Varicella: encephalitis (excluding cerebellitis), hepatitis, pneumonia**	Herpes viral infections
Vibriosis (invasive disease due to Vibrio vulnificus)	Vibrio infections
**West Nile, Usutu: chronic encephalitis**	Flaviviral infections

In bold, those pathogens/presentations modified by the Safety Adjudication Committee (SAC) after consensus and literature review on the basis of Winthrop et al.’s paper [32]. *PVAN* polyomavirus-associated nephropathy, *BAL* bronchoalveolar lavage, *CNS* central nervous system, *CSF* cerebrospinal fluid, *DMARD* disease-modifying anti-rheumatic drug, *CMV* cytomegalovirus

### Step 2: Retrieval of infections in Pharmachild

Among the 8274 patients enrolled in the Pharmachild registry as of January 2017, 895 (10.8%) patients had experienced 1585 infections. A total of 772 events (48.7%) in 572 patients (Fig. 1 and step 3 of the “Methods” section) were eligible for the evaluation by the SAC, of which 437 were defined as preliminary OI, as per the provisional list of opportunistic pathogens/presentations, and 335 as very severe/severe or serious non-OI events (Fig. 1).

The baseline characteristics of the 572/895 (63.9%) adjudicated patients are reported in Table 2 in comparison with those who were not adjudicated. Among the 895 patients with infections, about 85% were from Europe, specifically 29.3% from Italy and 23.6% from the Netherlands, while the remaining patients were distributed among Russia (8%), South America (4%), Middle East, and India (3%). The adjudicated group was represented by younger patients, with longer disease duration, higher frequency of systemic JIA, and more frequent use of systemic glucocorticoids.

**Table 2:** Demographic and clinical characteristics of the Pharmachild patients with infections

**Data are*n*(%) or medians with IQR range**	Patients adjudicated* (*N* = 572)	Patients not adjudicated* (*N* = 323)	**Patients with infections (*N* = 895)**	***P*(patients adjudicated vs not adjudicated)**	
**Females**	388 (67.8%)	241 (74.6%)	629 (70.3%)	0.033	
**Age at onset**	3.1 (1.7–6.7)	4.1 (2.1–8.5)	3.5 (1.9–7.3)	0.001	
**Age at JIA diagnosis**	3.7 (2.1–7.5)	4.9 (2.4–9.5)	4.1 (2.2–8.1)	0.001	
**Disease duration at last FU**	7.6 (5.0–11.1)	5.8 (3.1–10.3)	7.1 (4.2–10.8)	< 0.001	
**JIA category**				0.004	
**Systemic**	120 (20.9%)	37 (11.4%)	157 (17.5%)		
**Oligo persistent**	101 (17.7%)	80 (24.8%)	181 (20.2%)		
**Oligo extended**	100 (17.5%)	50 (15.5%)	150 (16.8%)		
**Polyarticular RF-**	132 (23.1%)	84 (26.0%)	216 (24.1%)		
**Polyarticular RF+**	19 (3.3%)	15 (4.6%)	34 (3.8%)		
**Psoriatic**	25 (4.4%)	8 (2.5%)	33 (3.7%)		
**Enthesitis**	36 (6.3%)	21 (6.5%)	57 (6.4%)		
**Undifferentiated**	39 (6.8%)	28 (8.7%)	67 (7.5%)		
**Systemic glucocorticoids**	336 (58.7%)	154 (47.7%)	490 (54.7)	0.001	
**Synthetic DMARDs**					
**Methotrexate**	532 (93.0%)	289 (89.5%)	821 (91.7%)	0.065 < 0.001	
**Cyclosporine**	90 (15.7%)	13 (4.1%)	103 (11.5%)	< 0.001	
**Sulfasalazine**	66 (11.5%)	28 (8.7%)	94 (10.5%)	0.179	
**Leflunomide**	40 (7.0%)	28 (8.7%)	68 (7.6%)	0.364	
**Azathioprine**	17 (3.0%)	6 (1.9%)	23 (2.6%)	0.312	
**Hydroxychloroquine**	14 (2.4%)	9 (2.8%)	23 (2.6%)	0.758	
**Thalidomide**	7 (1.2%)	2 (0.6%)	9 (1.0%)	0.501	
**Biologic DMARDs**					
**Etanercept**	298 (52.1%)	126 (39.0%)	424 (47.4%)	< 0.001	
**Adalimumab**	178 (31.1%)	82 (25.4%)	260 (29.1%)	0.070	
**Tocilizumab**	103 (18.0%)	19 (5.9%)	122 (13.6%)	< 0.001	
**Infliximab**	84 (14.7%)	17 (5.3%)	101 (11.3%)	< 0.001	
**Anakinra**	54 (9.4%)	28 (8.7%)	82 (9.2%)	0.701	
**Abatacept**	39 (6.8%)	17 (5.3%)	56 (6.3%)	0.356	
**Canakinumab**	28 (4.9%)	10 (3.1%)	38 (4.2%)	0.200	
**Rituximab**	26 (4.5%)	3 (0.9%)	29 (3.2%)	0.003	
**Golimumab**	14 (2.4%)	6 (1.9%)	20 (2.2%)	0.566	
**Certolizumab**	4 (0.7%)	1 (0.3%)	5 (0.6%)	0.453	
**Other biologic agents**	2 (0.3%)	1 (0.3%)	3 (0.3%)	1.000	

Data are *n* (%) or medians with IQR range. Drugs refer to their administration at any time during the patient’s history and are sorted by their descending frequencies. *The adjudicated patients are represented by those with opportunistic infections as per the provisional list of opportunistic pathogens/presentations (step 1), and very severe/severe or serious non-opportunistic infections. The remaining ones represent the not adjudicated patients. *JIA* juvenile idiopathic arthritis, *FU* follow-up, *RF* rheumatoid factor, *DMARDs* disease-modifying anti-rheumatic drugs

### Step 3: Adjudication of infections by the SAC

A total of 689/772 (89.2%) events achieved consensus (3/5 SAC members) on the first 3 adjudication questions, and of these, 682 (99.0%) were considered as infections by the SAC and included in the analysis (Table 3). The majority of the 682 infections were considered common (88.4%), with 119 (17.4%) classified as opportunistic by the SAC after evaluation of the whole patient’s history. The consensus on the last 2 questions was more difficult to reach. Regarding the fourth question on the appropriateness of the treatment for the infection, consensus was achieved for 484 (77.1%) events, while for 140 (22.3%) of the cases, it was impossible to determine the suitability of the anti-infective treatment.

**Table 3:** Frequency of answers by the SAC. Consensus by the majority of the Safety Adjudication Committee (SAC) members (3/5) was required on the first 3 questions, so that 689 events were adjudicated by the panel. Among them, 682 were confirmed as infections and retained for the analysis

**Question for adjudication by the SAC**	**Yes**	**No**	**Impossible to determine**	**Total with consensus**
**1. Based on the information provided, do you confirm that this patient had an infection?**	682 (99%)	0	7 (1%)	689 (100%)
**2. Is this infection common?**	603 (88.4%)	78 (11.4%)	1 (0.2%)	682 (100%)
**3. Is this an opportunistic infection?**	119 (17.4%)	556 (81.5%)	7 (1%)	682 (100%)
**4. Was the treatment appropriate for the infection?**	484 (77.1%)	4 (0.6%)	140 (22.3%)	628 (92%)
**5. Could the event be possibly related to any of the drug(s) taken at the time of the event?**	307 (76.2%)	70 (17.4%)	2 (0.5%)	403^*^ (59%)

*n = 24 were events without answers for the lack of consensus by the panel (less than 3/5 experts agreeing on the answer)

Similarly, for the fifth question about the possible relationship between the infection and the related treatment(s) for JIA, the lack of consensus increased up to 279 (41%). For 307/403 (76.2%) cases for which there was consensus, the SAC considered the drug(s) possibly related to the event. When we considered the drugs administered at the time of infection, the association of 1 biologic (more commonly etanercept or adalimumab) plus 1 synthetic DMARD was the most frequently reported (32% of the cases), followed by methotrexate alone (21%) and etanercept alone (20.3%), and finally by the association of either 1 biologic plus 1 synthetic DMARD plus systemic glucocorticoids (9%) or 1 synthetic DMARD plus systemic glucocorticoids (3.7%).

### Step 4: Analysis of the infections according to MedDRA dictionary

The evaluation of the Pharmachild registry conducted by the SAC led to the adjudication of the 682 infections corresponding to 53 HLTs and 153 PTs. For 92 (60%) PTs, the expert committee confirmed the same PT used by the Pharmachild Medical Monitor, while for the remaining 40%, discrepancies were solved by the study SC after re-evaluation of the individual cases. The final number of HLTs was 50, with corresponding 149 PTs, showed in details with the frequency of the events in the additional table 1.

### Step 5: Final evidence-based listing of opportunistic pathogens/infection presentations

After matching the adjudicated events with the provisional list of OI, among the 682 events, 106 (15.5%) for 22 PT were classified as “confirmed OI,” 274 (40.2%) for 89 PT were classified as “confirmed non-OI,” and 302 (44.3%) for 38 PT were classified as “possible/patient and/or pathogen- related OI.”

Table 4 shows the frequency of the 106 “confirmed OI” by HLT/PT in 93 patients. Regarding pathogens, herpes viral infections resulted the most frequent HLT/PT category, with 72 events (68% of the total confirmed OI), mostly represented by herpes zoster infection (66/72, 91.6%). Among the 64 patients with 72 confirmed herpes zoster infections, 35/64 (54.7%) had varicella in the past history and later developed herpes zoster (34 patients) and herpes zoster oticus (1 patient). One out of 35 patients, who had been vaccinated against varicella zoster had varicella in the past, and later zoster infection. Two additional patients had been vaccinated for varicella among those who developed zoster infection without having varicella reported in the history. Tuberculosis, *Candida*, papilloma, and *Pneumocystis* followed with a frequency higher than 3%. Among the 4 papilloma viral infections, affecting 2 patients in their history, no one was preceded by HPV vaccination. Of all the 29 *Mycobacterium tuberculosis* infections in Pharmachild (additional table 1), only 11/29 (38%) were “confirmed OI,” mostly in female patients (70%), at a median age of 5.2 years, not previously vaccinated with BCG, with pulmonary or disseminated presentations. The remaining were either latent tuberculosis or not well-specified contact with the pathogen, classified by the SAC as “possible/patient and/or pathogen-related OI.” The majority of the “confirmed OI” was reported in Europe (75.5%), while 11.3% was reported in Russia, 9.4% in Brazil, and 1.9% in India and Israel. These events occurred after a median period of 5.3 years from disease onset (IQR 3.4–9.2). Scanty data were reported on the immune status of the patients with “confirmed OI” at the moment of infection and soon afterwards. For 17.8% (10/93) of the patients with “confirmed OI,” there was evidence of lymphocytes below 500/μl only in 2 patients with cytomegalovirus and herpes zoster infection. No other immunological abnormalities could be observed (data not shown).

**Table 4:** Frequency of the 106 infections classified as “confirmed OI” by the SAC. Opportunistic infections (OI) were classified as “confirmed OI” after the evaluation of the cases available in Pharmachild with full agreement between the Safety Adjudication Committee (SAC) and the list of provisional pathogens/presentations. Data are presented as per the MedDRA High Level and Preferred Term and sorted by frequencies in descending order

**HLT-PT name**	**“Confirmed OI”** ***N*** **= 106**	**Patients** ***N*** = 93*
**Herpes viral infections**	**72 (68%)**	**64 (68.8%)**
Herpes zoster	66 (91.6%)	
Herpes ophthalmic	2 (2.8%)	
Ophthalmic herpes zoster	2 (2.8%)	
Herpes virus infection	1 (1.4%)	
Herpes zoster oticus	1 (1.4%)	
**Tuberculous infections**	**11 (10.4%)**	**10 (10.8%)**
Pulmonary tuberculosis	6 (54.5%)	
Disseminated tuberculosis	4 (36.4%)	
Bone tuberculosis	1 (9.1%)	
***Candida*infections**	**9 (8.5%)**	**9 (9.7%)**
Oral candidiasis	4 (44.4%)	
*Candida* pneumonia	2 (22.2%)	
Balanitis candida	1 (11.1%)	
*Candida* sepsis	1 (11.1%)	
Esophageal candidiasis	1 (11.1%)	
**Papilloma viral infections**	**4 (3.8%)**	**2 (2.2%)**
Vulvovaginal human papilloma virus infection	3 (75%)	
Anogenital warts	1 (25%)	
***Pneumocystis*infections**	**4 (3.8%)**	**4 (4.3%)**
*Pneumocystis jirovecii* pneumonia	4 (100%)	
**Cytomegaloviral infections**	**3 (2.8%)**	**3 (3.2%)**
Cytomegalovirus mononucleosis	1 (33.3%)	
Cytomegalovirus viraemia	1 (33.3%)	
Pneumonia cytomegaloviral	1 (33.3%)	
**Aspergillus infections**	**1 (0.9%)**	**1 (1.1%)**
Bronchopulmonary aspergillosis	1 (100%)	
**Leprous infections**	**1 (0.9%)**	**1 (1.1%)**
Leprosy	1 (100%)	
**Infections NEC**	**1 (0.9%)**	**1 (1.1%)**
Infection in an immunocompromised host	1 (100%)	

*One patient may have been suffering from different OI over time

When we considered the most frequent “confirmed OI,” namely herpes zoster infections, *Candida* infections, and HPV infections, we noticed that patients were mostly female, with a median age at event onset between 5 and 6 years, except for HPV infection, with a median age at the event onset during adolescence (median 14.5 years, IQR 11.9–17.1).

The most frequent “confirmed OI,” herpes zoster and tuberculosis, occurred in the majority of the cases, during treatment with biologics (70.8% and 90.9%, respectively) and methotrexate (56.9% and 90.9%, respectively), followed by systemic glucocorticoids (19.4% and 27.3%, respectively). For *Candida*, glucocorticoids were reported in half of the cases, followed by biologics. By excluding one patient who got one steroid pulse at high dose and developed disseminated tuberculosis, the remaining patients with “confirmed OI” received a median dose of prednisone of 15 mg/day concomitantly to the infection. Details on the remaining infections can be found in the additional table 2.

Table 5 reports the frequency of “confirmed non-OI” and “possible/patient and/or pathogen-related OI,” after removing 218 infections for which PTs did not include a specific pathogen (the complete list of “confirmed non-OI” and “possible/patient and/or pathogen- related OI” is presented in additional table 1). Among the 274 infections classified as “confirmed non-OI,” only 59 (21.5%) were related to a specific pathogen, most frequently influenza virus, *Streptococcus*, *Staphylococcus*, and *Escherichia*. Conversely, almost all the infections classified as “possible/patient and/or pathogen-related OI” (299/302, 99%) were related to a specific pathogen.

**Table 5:** Frequency of the “confirmed non-OI” and “possible/patient and pathogen-related OI” adjudicated by the SAC

**HLT-PT name** ***N*(%)**	**“Confirmed non-OI”** ***N*** **= 59 (%)**	**“Possible/patient and pathogen-related OI”** ***N*** **= 299 (%)**
**Herpes viral infections,*N*** **= 193 (64.5)**		
Varicella		128 (42.8)
Oral herpes		30 (10.1)
Varicella zoster virus infection		24 (8.1)
Other herpes infections		11 (3.7)
**Epstein-Barr viral infections,*N*** **= 38 (12.7)**		
Epstein-Barr virus infection		22 (7.4)
Other EBV infections		16 (5.3)
**Tuberculous infections,*N*** **= 18 (6.0)**		
Latent tuberculosis		12 (4.1)
Tuberculosis		6 (2.0)
***Candida*infections,*N*** **= 8 (2.7)**		
Vulvovaginal candidiasis		6 (2.1)
Other candidiasis		2 (0.6)
**Influenza viral infections,*N*** **= 14 (23.7)**		
Influenza	13 (22)	
H1N1 influenza	1 (1.7)	
**Streptococcal infections,*N*** **= 14 (23.7)**		
Scarlet fever	4 (6.7)	
Pharyngitis streptococcal	3 (5.1)	
Other streptococcal infections	7 (11.9)	
***Salmonella*infections,*N*** **= 9 (3.0)**		
Gastroenteritis salmonella		6 (2.1)
Other *Salmonella* infections		3 (1.0)
**Molluscum contagiosum viral infections,*N*** **= 7(2.3) (2.3)**		
Molluscum contagiosum		7 (2.3)
**Staphylococcal infections,*N*** **= 5 (8.5)**		
Staphylococcal sepsis	2 (3.4)	
Other staphylococcal infections	3 (5.1)	
***Escherichia *infections,*N*** **= 4 (6.8)**		
*Escherichia* pyelonephritis	3 (5.1)	
Cystitis *Escherichia*	1 (1.7)	
**Skin structures and soft tissue infections,*N*** **= 3 (5.1) (5.1)**		
Impetigo	3 (5.1)	
***Bordetella *infections**, ***N*** **= 3 (5.1)**		
Pertussis	2 (3.4)	
*Bordetella* infection	1 (1.7)	
***Mycoplasma infections, N*** **= 3 (5.1)**		
*Mycoplasma* infections	3 (5.1)	
***Yersinia infections, N *= 2 (3.4)**		
Gastroenteritis *yersinia*	2 (3.4)	
**Other infections (HLT frequency < 2%)**	11 (18.6)	26 (8.7)

Clinical presentations were removed because of the lack of the specified pathogen. Data are presented as per the MedDRA High Level Term and Preferred Term and sorted by frequencies in descending order. Only pathogens with HLT % ≥ 2% are presented in details. The full listing is available in additional table 1. *SAC* Safety Adjudication Committee

Most of the herpes virus infections (193/299, 64.5%) were classified as “possible patient- and/or pathogen-related OI” with a different clinical presentation compared to the previous group of “confirmed OI.” In particular, varicella was the most common herpetic manifestation in this group, with 155/299 (51.8%) cases, followed by herpes simplex infections. Epstein-Barr virus infections were reported in 38/299 cases (12.7%), classified as infectious mononucleosis in 13 cases (4.3%). Latent tuberculosis accounted for 12/299 (4.1%) cases, followed by a few cases of tuberculosis, also with lymph node involvement included in this group. The remaining events of “possible/patient and/or pathogen-related OI” affected less than 3% of the cases.

## Discussion

An evidence-based list of opportunistic pathogens with the related MedDRA classification in immunosuppressed children with JIA has been derived by the combination of consensus among a panel of pediatricians with expertise in rheumatology and infectious diseases, and the analysis of the Pharmachild international registry in JIA [39]. The final list of opportunistic infections/presentations could constitute a future reference for researchers, pharmaceutical companies, and regulatory authorities dealing with pharmacovigilance issues.

The introduction of biologics in the 2000s for the treatment of JIA has dramatically changed the prognosis of children affected by JIA, but has also raised concerns on the possible risk of infections and other safety events in these patients. Despite the widespread use of these drugs, there is still a lack of knowledge regarding the assessment of the long-term safety of the biologics in JIA. In this context, the role of national and international registries becomes an important source of data [39, 45–47].

The Pharmachild international registry has the advantage of combining information from different countries based on real clinical data. In Pharmachild, infections occurred in about 11% of patients with JIA [39], and among them, it is of primary importance to identify the opportunistic infections that may impose a serious threat to the immunocompromised child. This is not an easy task, because apparently there is a large gap between what treating pediatric rheumatologists feel can be considered as an OI and what a panel of experts adjudicates as such. While most serious infections also occur in the healthy population, some events are more frequent or severe in case of immunosuppression. Conversely, some infections, such as tuberculosis, more common in immunocompromised children, may affect also the general population, although usually less severely [48]. Considering these difficulties in correctly defining OI, we made an effort to produce a document defining OI specifically in children with JIA on immunosuppression. Something similar was recently developed by a specialized Committee convened in the adult setting to define OI in adults and children with immune-mediated diseases on biologics [32]. With the same approach, our panel of specialists voted, through a three-step Delphi procedure, for a correct definition of definite and probable OI and subsequently produced a list of OI by cross-matching the provisional list produced by consensus with the Pharmachild data. In a first phase of our study, among the Pharmachild patients, a considerable percentage of infections (119/682, 17.4%) was adjudicated as opportunistic. When we matched the provisional list of OI with the patients’ clinical information, it became clear that other than events with full agreement between the SAC and the list, which could be considered either “confirmed OI” (106/682, 15.5%) or “non-confirmed OI” (274/682, 40.2%), there was a considerable number (299/682, 43.8%) of debatable infections due to the specific patient’s history and/or the pathogen presentation, and classified as “possible/patient and/or pathogen-related OI.” The best example is represented by herpes zoster (Tables 4 and 5). Varicella zoster presentation was included among the “confirmed OI,” as suggested in the majority of the literature in this issue [49–51]. However, primary varicella infection, frequently observed in our population (155/682, 22.7%), was included among the “possible/patient and/or pathogen-related OI” rather than “definite OI” due to the high incidence in healthy non-vaccinated children and its usually non-complicated presentation. This group of patients highlights the difficulties in defining OI in children with JIA on treatment, but also the critical importance of providing a reference document listing those infections that should always be considered as opportunistic in these patients, with possible implications for treatment or prophylaxis. Half of the patients with herpes zoster infections had varicella in their history indicating a possible subsequent reactivation of the virus due to a transient immunosuppressive condition. One patient developed varicella despite vaccination while 2 patients had herpes infection despite previous vaccination, without manifesting primary varicella. This observation may give rise to speculations about a possible increase in zoster infections in JIA population under immunosuppressive therapy through varicella infection as well as herpes zoster reactivation. Limited data are available on vaccinations for other infections such as papillomavirus, which occurred only in 2 patients not previously vaccinated. Therefore, it would be worthwhile to develop further studies focused specifically on this topic, in order to understand if vaccinations may maintain a protective immune status in JIA patients under treatment or not. It would also be interesting to investigate how to identify patients with JIA at risk for developing OI, but this would require further comparative studies on the immune status in JIA patients receiving different immunosuppressive treatments. Per definition, a definite OI can occur in patients without recognized forms of immunosuppression but its presence indicates a potential or likely alteration in host immunity. Therefore, it is worthwhile to consider each OI as relevant and representing a potential risk for the JIA patient’s life, thus requiring a prompt treatment. In fact, to the best of our knowledge, there are no studies indicating who is at major risk of complications due to an opportunistic pathogen among JIA patients on immunosuppressive therapy. The use of an immune screening to help primary care practitioners who may care for, diagnose, and manage infections is already consolidated in clinical practice [45]. In our study, we found no specific level of immunosuppression indicating an increased frequency of infections such as *Pneumocystis jirovecii*, although too little data are available on this issue and further analysis is needed to understand the correlation between immune status and OI in patients with autoimmune diseases.

Biologics and methotrexate were often seen at the time of a “confirmed OI.” Nevertheless, a comparative study about the role of immunosuppressive drugs would require a larger population and a deeper analysis, which was not the aim of the present manuscript.

Besides those pathogens confirmed as OI and non- or possible OI by the panel on the basis of the Pharmachild real patients’ data, there are also pathogens (e.g., *Nocardia*) that have been included in the list of definite/possible OI (Table 1) by consensus, although they were not identified in Pharmachild. These infections, apparently uncommon since there was none in such a large database, should be considered potential indicators of alterations in host immunity when present in JIA patients and deeply investigated by the physician of the center in order to prevent possible complications in these patients.

The current literature provides similar evidence, but remains controversial for the majority of OI. Beukelman et al. in 2012 reviewed US Medicaid data comparing the incidence of bacterial infections requiring hospitalization in children with and without JIA [1, 30]. The infection rate was already twice as high in patients with JIA not exposed to treatments, compared to children with attention-deficit hyperactivity disorder (ADHD) used as controls [30]. The same author 1 year later re-analyzed the same data by comparing the incidence rate of selected OI among children with and without JIA. *Coccidioides*, *Salmonella*, and herpes zoster were more common among children with JIA [31]. Among the 15 pathogens they used to define their list of OI, all in our provisional OI list (Table 1), only herpes zoster, tuberculosis, *Pneumocystis*, and *Aspergillus* were confirmed in our final list of “confirmed OI.” The remaining cases were included in the “possible/patient and/or pathogen-related OI” list. Interestingly, the authors included primary varicella infection in the OI only if the affected patient received critical care services during the hospitalization. An increased risk of herpes zoster infection was confirmed in many studies, both in JIA [49] and in adult rheumatoid arthritis [52]. More recently, Aeschlimann et al. studied, through a meta-analysis, whether treatment with biologics during clinical trial study periods increased the risk of serious infections in children with JIA. On a total of 19 trials accounting for 21 individual studies, 17 serious infections were reported among 810 children, with bronchopulmonary infections and varicella being the most frequent events [53]. Besides this evidence, the role of other opportunistic pathogens still needs to be further investigated, as well as the comparison of OI among large registries. Recently, Swart et al. have provided a comparison between Pharmachild and national registries. In particular, a comparable percentage of serious AE has been found between Pharmachild and the German registry Biker (6.9% and 7.4%, respectively), with an overlapping frequency of infection and infestations among all AEs (29.4–30.1%). Infections also resulted the most frequent ESI in both registries (75.3–89%). Interestingly, among OI, tuberculosis affected 27 cases in Pharmachild and none in BiKer, although this could be explained by the different geographic distribution of the patients [39].

A limitation of our study is that Pharmachild is mainly a European registry, although it includes countries worldwide. This means that our results mainly depict the European scenario of OI. A future manuscript will focus on those factors increasing the risk of OI through appropriate modeling to identify the risk factor for OI infection including disease duration, drugs, comorbidities, etc.

## Conclusions

In conclusion, almost 1/5 of all severe and/or serious infections in JIA patients on immunosuppressive therapy are opportunistic. The most frequent opportunistic pathogens were herpes virus (excluding non-complicated primary varicella), mycobacterial, and *Candida* infections. We provided with our work a list of “confirmed OI” in children with JIA on immunosuppressive therapy that could be used as possible reference document for future works on pharmacovigilance in children with JIA on immunosuppressive therapy and a list of infections that could possibly display an opportunistic nature related to the patient’s history and/or the pathogen presentation. More clarity in the understanding of OI in children with JIA on immunosuppressant will help in deciding on immunosuppressive treatment and prophylaxis in this group of patients.

## Supplementary information

**Additional file 1** figure. Flowchart of the process.

**Additional file 2 figure**. Hierarchy of MedDra clinically-validated international medical terminology.

**Additional file 3 Table 1.** Complete table with the frequency of the 682 infections adjudicated by the SAC. Infections were reported after evaluation of the cases available in Pharmachild compared to the pathogens/presentations in the provisional list approved by the Safety Adjudication Committee (SAC). Data are presented as per the MedDRA High Level Term (HLT) and Preferred Term (PT) sorted by frequencies in descending order (HLT and then PT). *For definition see Step 5.

**Additional file 4 Table 2.** Concomitant medications administered at the time of “confirmed OI”. Bio: biologic, mtx: methotrexate; ste: systemic steroids; sDMARDs: synthetic disease modifying antirheumatic drugs; *sDMARDs are intendend other than MTX.

## Data Availability

Pharmachild registry is registered at Clinicaltrials.gov (NCT01399281) and at the European Network of Centres for Pharmacoepidemiology and Pharmacovigilance (ENCePP; http://www.encepp.eu/encepp/viewResource.htm?id=19362).

## Abbreviations

OI: Opportunistic infections
JIA: Juvenile idiopathic arthritis
SAC: Safety Adjudication Committee
MedDRA: The Medical Dictionary for Regulatory Activities
DMARDs: Disease-modifying anti-rheumatic drugs
FDA: Food and Drug Administration
EMA: European Medicines Agency
PRINTO: Paediatric Rheumatology INternational Trials Organization
PReS: Paediatric Rheumatology European Society
AE: Adverse events
SAE: Serious adverse events
PT: Preferred term
SC: Steering Committee
SOC: System Organ Class
ESI: Events of Special Interest
SOP: Standard operating procedure
ILAR: International League Against Rheumatism
HLT: High Level Term
IQR: Inter-quartile range
ADHD: Attention-deficit hyperactivity disorder
