# Supplementary figures and images for: Opportunistic infections in immunosuppressed patients with juvenile idiopathic arthritis: analysis by the Pharmachild Safety Adjudication Committee
Source: Arthritis Res Ther. 2020 Apr 7;22:71. doi: 10.1186/s13075-020-02167-2 (PMC7136994; doi:10.1186/s13075-020-02167-2)

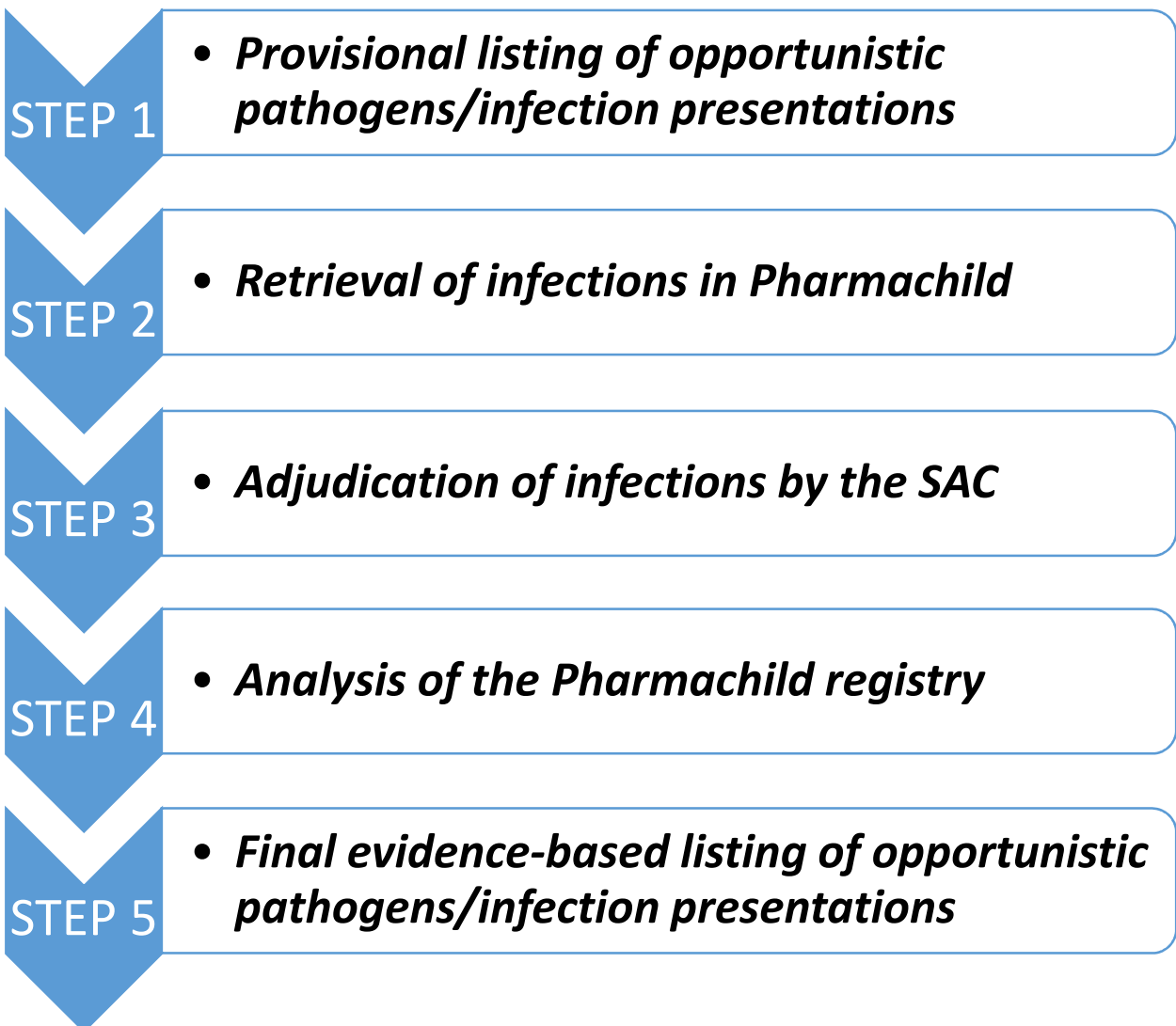

Supplement: Supplementary file 1 — Additional file 1 figure. Flowchart of the process. [file 13075_2020_2167_MOESM1_ESM.pdf]

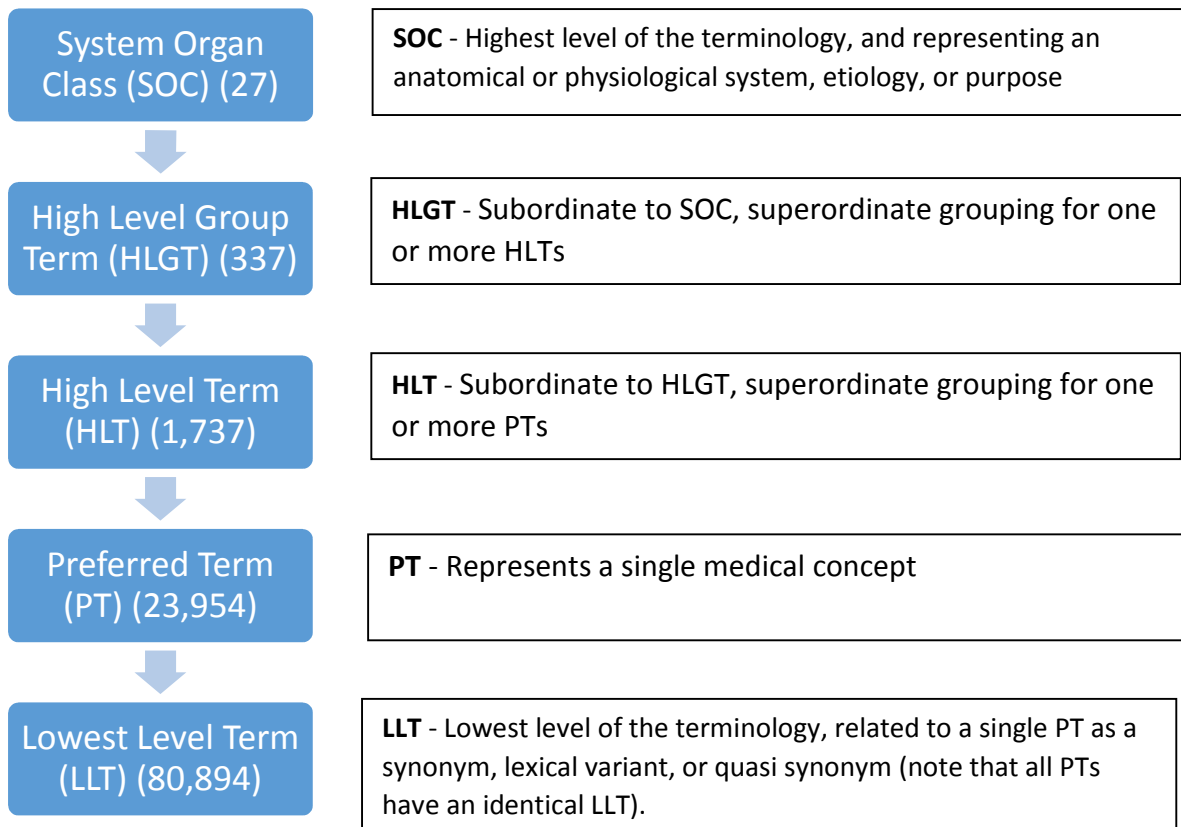

Supplement: Supplementary file 2 — Additional file 2 figure. Hierarchy of MedDra clinically-validated international medical terminology. [file 13075_2020_2167_MOESM2_ESM.pdf]
