# Supplementary material for: Opportunistic infections in immunosuppressed patients with juvenile idiopathic arthritis: analysis by the Pharmachild Safety Adjudication Committee
Source: Arthritis Res Ther. 2020 Apr 7;22:71. doi: 10.1186/s13075-020-02167-2 (PMC7136994; doi:10.1186/s13075-020-02167-2)
Supplement: Supplementary file 3 — Additional file 3 Table 1. Complete table with the frequency of the 682 infections adjudicated by the SAC. Infections were reported after evaluation of the cases available in Pharmachild compared to the pathogens/presentations in the provisional list approved by the Safety Adjudication Committee (SAC). Data are presented as per the MedDRA High Level Term (HLT) and Preferred Term (PT) sorted by frequencies in descending order (HLT and then PT). *For definition see Step 5. [file 13075_2020_2167_MOESM3_ESM.docx]

**Additional Table 1. Complete table with the frequency of the 682 infections adjudicated by the SAC.** Infections were reported after evaluation of the cases available in Pharmachild compared to the pathogens/presentations in the provisional list approved by the Safety Adjudication Committee (SAC). Data are presented as per the MedDRA High Level Term (HLT) and Preferred Term (PT) sorted by frequencies in descending order (HLT and then PT). *For definition see Step 5.

| **HLT-PT NAME** | **N** | **%** | **“Confirmed OI”*** | **“Confirmed Non-OI”*** | “**Possible/patient and/or pathogen related OI**” ***** |
| --- | --- | --- | --- | --- | --- |
| **Herpes viral infections** | **265** | **38.9%** |  |  |  |
| Varicella | 128 | 48.3% |  |  | x |
| Herpes zoster | 66 | 24.9% | x |  |  |
| Oral herpes | 30 | 11.3% |  |  | x |
| Varicella zoster virus infection | 24 | 9.1% |  |  | x |
| Herpes simplex | 4 | 1.5% |  |  | x |
| Varicella zoster pneumonia | 3 | 1.1% |  |  | x |
| Herpes ophthalmic | 2 | 0.7% | x |  |  |
| Exanthema subitum | 1 | 0.4% |  |  | x |
| Genital herpes simplex | 1 | 0.4% |  |  | x |
| Herpes dermatitis | 1 | 0.4% |  |  | x |
| Herpes virus infection | 1 | 0.4% | x |  |  |
| Herpes zoster oticus | 1 | 0.4% | x |  |  |
| Ophthalmic herpes simplex | 1 | 0.4% |  |  | x |
| Ophthalmic herpes zoster | 2 | 0.7% | x |  |  |
| **Lower respiratory tract and lung infections** | **49** | **7.2%** |  |  |  |
| Pneumonia | 41 | 83.6% |  | x |  |
| Atypical pneumonia | 2 | 4.1% |  | x |  |
| Bronchitis | 2 | 4.1% |  | x |  |
| Infectious pleural effusion | 2 | 4.1% |  |  | x |
| Lower respiratory tract infection | 2 | 4.1% |  | x |  |
| **Upper respiratory tract infections** | **44** | **6.4%** |  |  |  |
| Upper respiratory tract infection | 14 | 31.8% |  | x |  |
| Tonsillitis | 11 | 25% |  | x |  |
| Pharyngitis | 8 | 18.2% |  | x |  |
| Sinusitis | 3 | 6.8% |  | x |  |
| Chronic sinusitis | 2 | 4.5% |  | x |  |
| Pharyngotonsillitis | 2 | 4.5% |  | x |  |
| Rhinitis | 2 | 4.5% |  | x |  |
| Adenoiditis | 1 | 2.3% |  | x |  |
| Laryngitis | 1 | 2.3% |  | x |  |
| **Epstein-Barr viral infections** | **38** | **5.6%** |  |  |  |
| Epstein-Barr virus infection | 22 | 57.9% |  |  | x |
| Infectious mononucleosis | 13 | 34.2% |  |  | x |
| Epstein-Barr viraemia | 2 | 5.3% |  |  | x |
| Hepatitis infectious mononucleosis | 1 | 2.6% |  |  | x |
| **Abdominal and gastrointestinal infections** | **32** | **4.7%** |  |  |  |
| Gastroenteritis | 15 | 46.9% |  | x |  |
| Appendicitis | 12 | 37.5% |  | x |  |
| Appendicitis perforated | 3 | 9.4% |  | x |  |
| Anal abscess | 1 | 3.1% |  | x |  |
| Gastrointestinal infection | 1 | 3.1% |  | x |  |
| **Tuberculous infections** | **29** | **4.2%** |  |  |  |
| Latent tuberculosis | 12 | 41.4% |  |  | x |
| Pulmonary tuberculosis | 6 | 20.7% | x |  |  |
| Disseminated tuberculosis | 4 | 13.8% | x |  |  |
| Tuberculosis | 3 | 10.3% |  |  | x |
| Tuberculosis of intrathoracic lymph nodes | 3 | 10.3% |  |  | x |
| Bone tuberculosis | 1 | 3.4% | x |  |  |
| **Bacterial infections NEC** | **27** | **4%** |  |  |  |
| Pneumonia bacterial | 11 | 40.8% |  | x |  |
| Cellulitis | 2 | 7.4% |  | x |  |
| Nail bed infection bacterial | 2 | 7.4% |  | x |  |
| Upper respiratory tract infection bacterial | 2 | 7.4% |  | x |  |
| Urinary tract infection bacterial | 2 | 7.4% |  | x |  |
| Wound infection bacterial | 2 | 7.4% |  | x |  |
| Ear infection bacterial | 1 | 3.7% |  | x |  |
| Lymphadenitis bacterial | 1 | 3.7% |  | x |  |
| Otitis externa bacterial | 1 | 3.7% |  | x |  |
| Peritonitis bacterial | 1 | 3.7% |  | x |  |
| Pharyngitis bacterial | 1 | 3.7% |  | x |  |
| Pyomyositis | 1 | 3.7% |  | x |  |
| **Infections NEC** | **21** | **3.1%** |  |  |  |
| Respiratory tract infection | 12 | 57.1% |  | x |  |
| Abscess limb | 2 | 9.5% |  | x |  |
| Postoperative wound infection | 2 | 9.5% |  | x |  |
| Wound infection | 2 | 9.5% |  | x |  |
| Infection in an immunocompromised host | 1 | 4.8% | x |  |  |
| Injection site infection | 1 | 4.8% |  | x |  |
| Lymph node abscess | 1 | 4.8% |  | x |  |
| **Ear infections** | **18** | **2.6%** |  |  |  |
| Otitis media acute | 8 | 44.4% |  | x |  |
| Otitis media | 5 | 27.7% |  | x |  |
| Ear infection | 3 | 16.7% |  | x |  |
| Otitis externa | 1 | 5.6% |  | x |  |
| Otitis media chronic | 1 | 5.6% |  | x |  |
| **Candida infections** | **17** | **2.5%** |  |  |  |
| Vulvovaginal candidiasis | 6 | 35.3% |  |  | x |
| Oral candidiasis | 4 | 23.5% | x |  |  |
| Candida pneumonia | 2 | 11.7% | x |  |  |
| Anal candidiasis | 1 | 5.9% |  |  | x |
| Balanitis candida | 1 | 5.9% | x |  |  |
| Candida infection | 1 | 5.9% |  |  | x |
| Candida sepsis | 1 | 5.9% | x |  |  |
| Oesophageal candidiasis | 1 | 5.9% | x |  |  |
| **Influenza viral infections** | **14** | **2.1%** |  |  |  |
| Influenza | 13 | 92.9% |  | x |  |
| H1N1 influenza | 1 | 7.1% |  | x |  |
| **Streptococcal infections** | **14** | **2.1%** |  |  |  |
| Scarlet fever | 4 | 28.6% |  | x |  |
| Pharyngitis streptococcal | 3 | 21.4% |  | x |  |
| Erysipelas | 2 | 14.3% |  | x |  |
| Pneumonia pneumococcal | 2 | 14.3% |  | x |  |
| Streptococcal bacteraemia | 1 | 7.1% |  | x |  |
| Streptococcal infection | 1 | 7.1% |  | x |  |
| Streptococcal sepsis | 1 | 7.1% |  | x |  |
| **Salmonella infections** | **9** | **1.3%** |  |  |  |
| Gastroenteritis salmonella | 6 | 66.7% |  |  | x |
| Salmonella bacteraemia | 1 | 11.1% |  |  | x |
| Salmonellosis | 1 | 11.1% |  |  | x |
| Typhoid fever | 1 | 11.1% |  |  | x |
| **Urinary tract infections** | **9** | **1.3%** |  |  |  |
| Pyelonephritis | 5 | 55.6% |  | x |  |
| Urinary tract infection | 2 | 22.2% |  | x |  |
| Cystitis | 1 | 11.1% |  | x |  |
| Pyelonephritis acute | 1 | 11.1% |  | x |  |
| **Cytomegaloviral infections** | **8** | **1.2%** |  |  |  |
| Cytomegalovirus infection | 5 | 62.5% |  |  | x |
| Cytomegalovirus mononucleosis | 1 | 12.5% | x |  |  |
| Cytomegalovirus viraemia | 1 | 12.5% | x |  |  |
| Pneumonia cytomegaloviral | 1 | 12.5% | x |  |  |
| **Molluscum contagiosum viral infections** | **7** | **1.1%** |  |  |  |
| Molluscum contagiosum | 7 | 100% |  |  | x |
| **Papilloma viral infections** | **7** | **1.1%** |  |  |  |
| Papilloma viral infection | 3 | 42.8% |  |  | x |
| Vulvovaginal human papilloma virus infection | 3 | 42.8% | x |  |  |
| Anogenital warts | 1 | 14.4% | x |  |  |
| **Sepsis, bacteraemia, viraemia and fungaemia NEC** | **7** | **1.1%** |  |  |  |
| Device related sepsis | 2 | 28.6% |  | x |  |
| Sepsis | 2 | 28.6% |  | x |  |
| Sepsis syndrome | 2 | 28.6% |  | x |  |
| Viraemia | 1 | 14.3% |  |  | x |
| **Campylobacter infections** | **5** | **0.7%** |  |  |  |
| Campylobacter gastroenteritis | 5 | 100% |  |  | x |
| **Staphylococcal infections** | **5** | **0.7%** |  |  |  |
| Staphylococcal sepsis | 2 | 40% |  | x |  |
| Furuncle | 1 | 20% |  | x |  |
| Pneumonia staphylococcal | 1 | 20% |  | x |  |
| Toxic shock syndrome staphylococcal | 1 | 20% |  | x |  |
| **Viral infections NEC** | **5** | **0.7%** |  |  |  |
| Viral upper respiratory tract infection | 3 | 60% |  | x |  |
| Gastroenteritis viral | 2 | 40% |  | x |  |
| **Escherichia infections** | **4** | **0.6%** |  |  |  |
| Escherichia pyelonephritis | 3 | 75% |  | x |  |
| Cystitis escherichia | 1 | 25% |  | x |  |
| **Pneumocystis infections** | **4** | **0.6%** |  |  |  |
| Pneumocystis jirovecii pneumonia | 4 | 100% | x |  |  |
| **Skin structures and soft tissue infections** | **4** | **0.6%** |  |  |  |
| Impetigo | 3 | 75% |  | x |  |
| Subcutaneous abscess | 1 | 25% |  | x |  |
| **Bordetella infections** | **3** | **0.4%** |  |  |  |
| Pertussis | 2 | 66.7% |  | x |  |
| Bordetella infection | 1 | 33.3% |  | x |  |
| **Dental and oral soft tissue infections** | **3** | **0.4%** |  |  |  |
| Tooth abscess | 2 | 66.7% |  | x |  |
| Sialoadenitis | 1 | 33.3% |  | x |  |
| **Giardia infections** | **3** | **0.4%** |  |  |  |
| Giardiasis | 3 | 100% |  |  | x |
| **Mycoplasma infections** | **3** | **0.4%** |  |  |  |
| Mycoplasma infection | 1 | 33.3% |  | x |  |
| Pharyngitis mycoplasmal | 1 | 33.3% |  | x |  |
| Pneumonia mycoplasmal | 1 | 33.3% |  | x |  |
| **Caliciviral infections** | **2** | **0.3%** |  |  |  |
| Gastroenteritis caliciviral | 1 | 50% |  | x |  |
| Gastroenteritis norovirus | 1 | 50% |  |  | x |
| **Eye and eyelid infections** | **2** | **0.3%** |  |  |  |
| Conjunctivitis | 2 | 100% |  | x |  |
| **Hepatitis viral infections** | **2** | **0.3%** |  |  |  |
| Hepatitis B | 1 | 50% |  |  | x |
| Hepatitis C | 1 | 50% |  |  | x |
| **Parvoviral infections** | **2** | **0.3%** |  |  |  |
| Parvovirus B19 infection | 2 | 100% |  |  | x |
| **Rotaviral infections** | **2** | **0.3%** |  |  |  |
| Gastroenteritis rotavirus | 2 | 100% |  |  | x |
| **Yersinia infections** | **2** | **0.3%** |  |  |  |
| Gastroenteritis yersinia | 1 | 50% |  | x |  |
| Yersinia infection | 1 | 50% |  | x |  |
| **Aspergillus infections** | **1** | **0.1%** |  |  |  |
| Bronchopulmonary aspergillosis | 1 | 100% | x |  |  |
| **Blastocystis infections** | **1** | **0.1%** |  |  |  |
| Blastocystis infection | 1 | 100% |  | x |  |
| **Bone and joint infections** | **1** | **0.1%** |  |  |  |
| Osteomyelitis acute | 1 | 100% |  | x |  |
| **Borrelial infections** | **1** | **0.1%** |  |  |  |
| Lyme disease | 1 | 100% |  | x |  |
| **Clostridia infections** | **1** | **0.1%** |  |  |  |
| Clostridium difficile colitis | 1 | 100% |  | x |  |
| **Coxiella infections** | **1** | **0.1%** |  |  |  |
| Coxiella infection | 1 | 100% |  | x |  |
| **Enteroviral infections NEC** | **1** | **0.1%** |  |  |  |
| Enterovirus infection | 1 | 100% |  |  | x |
| **Fungal infections NEC** | **1** | **0.1%** |  |  |  |
| Systemic mycosis | 1 | 100% |  |  | x |
| **Haemophilus infections** | **1** | **0.1%** |  |  |  |
| Haemophilus infection | 1 | 100% |  | x |  |
| **Helicobacter infections** | **1** | **0.1%** |  |  |  |
| Helicobacter gastritis | 1 | 100% |  | x |  |
| **Leprous infections** | **1** | **0.1%** |  |  |  |
| Leprosy | 1 | 100% | x |  |  |
| **Muscle and soft tissue infections** | **1** | **0.1%** |  |  |  |
| Psoas abscess | 1 | 100% |  | x |  |
| **Mycobacteria identification and serology** | **1** | **0.1%** |  |  |  |
| Tuberculin test positive | 1 | 100% |  | x |  |
| **Pseudomonal infections** | **1** | **0.1%** |  |  |  |
| Pseudomonal sepsis | 1 | 100% |  | x |  |
| **Respiratory syncytial viral infections** | **1** | **0.1%** |  |  |  |
| Respiratory syncytial virus infection | 1 | 100% |  |  | x |
| **Rubeola viral infections** | **1** | **0.1%** |  |  |  |
| Pneumonia measles | 1 | 100% |  | x |  |
