# Supplementary material for: Opportunistic infections in immunosuppressed patients with juvenile idiopathic arthritis: analysis by the Pharmachild Safety Adjudication Committee
Source: Arthritis Res Ther. 2020 Apr 7;22:71. doi: 10.1186/s13075-020-02167-2 (PMC7136994; doi:10.1186/s13075-020-02167-2)
Supplement: Supplementary file 4 — Additional file 4 Table 2. Concomitant medications administered at the time of “confirmed OI”. Bio: biologic, mtx: methotrexate; ste: systemic steroids; sDMARDs: synthetic disease modifying antirheumatic drugs; *sDMARDs are intendend other than MTX. [file 13075_2020_2167_MOESM4_ESM.docx]

| **HLT-PT NAME** | **“Confirmed OI“**  **N=106** | **Concomitant medications** | **Concomitant medications per drug category** |
| --- | --- | --- | --- |
| **Herpes viral infections** | **72 (68%)** | 22 bio+mtx  16 bio  8 mtx  5 bio+mtx+ste  3 ste  2 bio +sDMARDS*  2 bio +sDMARDS*+mtx  2 bio+ ste  2 sDMARDs+mtx  1 sDMARDs+mtx+ste  1 sDMARDs+ste  1 bio +sDMARDS*+mtx+ste  1 bio +sDMARDS*+ste  6 other | 51 bio  41 mtx  14 ste  10 sDMARDs  6 other |
| **Tuberculous infections** | **11 (10.4%)** | 5 bio+mtx  2 bio+sDMARDs+mtx  1 mtx+ste  1 bio  1 bio+mtx+ste  1 bio+sDMARDs+mtx+ste | 10 bio  10 mtx  3 ste  3 sDMARDs |
| **Candida infections** | **9 (8.5%)** | 3 ste  3 bio+mtx  1 bio+ste  1 sDMARDs+ste  1 other | 5 ste  4 bio  3 mtx  1 other |
| **Papilloma viral infections** | **4 (3.8%)** | 3 mtx+ste  1 bio | 3 mtx  3 ste  1 bio |
| **Pneumocystis infections** | **4 (3.8%)** | 2 sDMARDs+mtx+ste  1 mtx  1 mtx+ste | 4 mtx  3 ste  2 sDMARDs |
| **Cytomegaloviral infections** | **3 (2.8%)** | 1 mtx  1 ste  1 bio+ste | 2 ste  1 bio  1 mtx |
| **Aspergillus infections** | **1 (0.9%)** | 1 ste | 1 ste |
| **Leprous infections** | **1 (0.9%)** | 1 bio | 1 bio |
| **Infections NEC** | **1 (0.9%)** | 1 ste | 1 ste |

Additional table 2. Concomitant medications administered at the time of “confirmed OI”. Bio: biologic, mtx: methotrexate; ste: systemic steroids; sDMARDs: synthetic disease modifying antirheumatic drugs; *sDMARDs are intendend other than MTX.
